# Supportive Transition Planning for Adolescents Transitioning From Psychiatric Hospitalization to School: A Systematic Literature Review and Framework of Practices

**DOI:** 10.5334/cie.61

**Published:** 2023-01-30

**Authors:** Sara Midura, Jill C. Fodstad, Benjamin White, Angela J. Turner, Scott Menner

**Affiliations:** 1 Northwest Education Services (current), US; 2 Riley Hospital for Children (former), US; 3 Indiana University Health; 4 Indiana University School of Medicine, US; 5 Multnomah ESD at The Unity Center for Behavioral Health, US; 6 Unity Center for Behavior Health, US; 7 Archdiocese of Cincinnati (Current), US; 8 Cincinnati Children’s Hospital (Former), US

**Keywords:** transition, education, hospital, psychiatric care, acute psychiatric care, behavioral health, hospital education

## Abstract

School-aged youth with behavioral health needs often struggle in the academic environment. When admitted to acute psychiatric hospital settings, the student’s difficulties and needs increase upon discharge and return to the school setting. While the literature describes systemic issues in transitioning from an acute psychiatric hospital to the school setting, limited resources exist for practitioners to plan for and support the successful reintegration of affected students. Using the Preferred Reporting Items for Systematic Reviews and Meta-Analyses (PRISMA) guidelines, the purpose of the current systematic review was to collect and synthesize evidence from the literature (*N* = 20) in the areas of barriers, challenges, and significance of the need for a formal transition planning framework. Four major key factors emerged as important to assist in creating a transition planning framework for acute psychiatric hospitals to school-based settings: (a) Stakeholder Voice (Student, Caregiver, Hospital/Treatment Team, or School Team Voice); (b) Establishing a Point Person for Transition (Medical or School Point Person); (c) Recommendations/Accommodations (Formal or Informal Supports); and (d) Having a Transition Meeting. Other common factors are discussed, and recommendations are provided to aid practitioners in increasing the likelihood that school-age youth succeed in the school environment post-discharge from acute psychiatric settings. Finally, gaps in the literature are identified as areas for further research.

Pediatric mental health is a public health crisis – one in five children and adolescents are diagnosed with a mental health disorder in the United States (National Research Council and Institute of Medicine, 2009). Over the last 25 years, the frequency of pediatric psychiatric service use (including acute inpatient psychiatric care) has increased by 81% for children and 42% for adolescents (Blader, 2011). As the demand for inpatient services have increased, the length of acute psychiatric hospitalizations has decreased (Blader, 2011). The juxtaposition of increased service need and reductions in length of stay presents problems in ensuring that enough time is provided to put appropriate supports in place for youth (i.e., children and adolescents) with psychiatric concerns.

Another factor affecting care for youth with acute psychiatric needs has been state education policy requirements for attendance accountability. Attendance policies require students to return to school almost immediately post-discharge (Clemens et al., 2011). As most insurance companies do not reimburse for school transition planning, students often return with minimal supports in place to maintain gains achieved during hospitalization (Blizzard et al., 2016; Tisdale, 2014). The barrier created by attendance accountability policies and insurance guidelines has resulted in the inability of caregivers (i.e., parent or other primary caregiver), students, and school staff to plan a supportive school transition. As a result, youth with mental health concerns are likely to experience difficulties with school engagement and academics (Cueller, 2015; McLeod et al., 2012). These difficulties are amplified when a student is transitioning back to school after an acute psychiatric hospitalization.

Finding ways to enhance collaboration between acute psychiatric hospitals and schools is necessary for students to successfully reintegrate. Frameworks and processes for transitioning youth with medical needs from hospitalization to school are well established (e.g., Bessell, 2001; Kaffenberger, 2006; Prevatt et al., 2000); however, similar literature for acute psychiatric hospitalization is sparse. While school transition frameworks and processes for medical hospitalizations for youth without psychiatric needs may inform psychiatric hospitalization transitions, certain considerations must occur for students with mental health diagnoses. Specifically, Simon and Savina (2005, 2010) suggest it is beneficial for schools to obtain disorder-specific information, to communicate with hospital personnel, and to receive a discharge summary, but for students transitioning from psychiatric hospitalizations, it is also critical to consider their mental health needs. Youth are at risk for rehospitalization during the transitional period, and when the student’s psychiatric needs are not prioritized, the likelihood for successful school reintegration is minimized (Simon & Savina, 2010; White et al., 2006). Against this background, the need for specific transition frameworks for youth admitted to acute psychiatric hospitalization is clear.

A few models exist for the successful transitioning back to school of students with acute psychiatric needs. For example, Savina and colleagues (2014) offered the first theoretical framework in the form of a template. According to their framework, key factors for transitions include considering multiple perspectives (e.g., patient, caregiver, school staff) and interdisciplinary collaboration. As a result, the template derived from their findings contains sections essential for the transition process: the youth’s needs, interventions to support the student, methods to support school staff, and relevant contact information.

Two program models for school transitions from acute psychiatric hospitalization also exist. Weiss et al. (2015) detail the School Transition Program within a pediatric psychiatric unit. Their model includes a designated school transition specialist and a family connector, who consult with families, hospital, and school staff to support reintegration needs through strategies such as daily check-ins, transition planning, peer-to-peer support, and family education. White et al. (2017) describe a model located in a community school setting, the Bridge for Resilient Youth in Transition (BRYT) program. The program provides focused interventions over 8–12 weeks post-discharge, including a dedicated transition classroom, a clinician to support coping, an academic interventionist to provide tutoring and help make up missed work, transition planning, coordination with other providers, and family support. The models proposed by Weiss et al. (2015) and White et al. (2017) are promising; however, their wide-scale adoption across settings is limited due to increased staffing needs and a consistent funding source. Future dissemination of the School Transition and BRYT programs is possible. However, an intermediate transition tool to bridge the gap between current resources and these future program expansions is needed.

Given the lack of clear and feasible guidelines, many schools and hospitals use Savina et al.’s (2014) framework to aid youth transitioning to school after psychiatric hospitalization. Unfortunately, researchers have questioned the validity of the methods and results of Savina et al. For example, Tougas et al. (2019) point out that Savina et al. do not provide a clear protocol on identifying, coding, and interpreting articles, which fails to achieve the criteria of transparency and reproducibility and, therefore, limits the scope of the work (Gough et al., 2012). Further, Savina et al. (2014) included other types of transitions to school (e.g., residential units, hospitalizations for chronic illness), thereby restricting the applicability of their outcomes and recommendations, including their template (Tougas et al., 2019).

Another concern with employing Savina et al.’s (2014) framework is that research in this field has grown in recent years and, therefore, their outcomes are outdated. In 2014, the literature on improving school transitions for youth discharged from acute psychiatric hospitalization was predominantly focused on or based on practitioner perspectives. Since then, the scope of research focus has widened to include other perspectives such as hospital-based providers (Clemens et al., 2010, 2011), student/patients (Iverson, 2017; Marraccini & Pittleman, 2021; Preyde et al., 2017; Preyde et al., 2021), school personnel (Marraccini et al., 2019; Marraccini et al., 2022), and caregivers (Blizzard et al., 2016).

Given the shortcomings of Savina et al.’s (2014) framework and the fact that it is the only tool published to date, it is necessary to develop an updated version. The new framework will create a focused and detailed process for hospital personnel and school staff to replicate in their efforts to achieve better transition outcomes for patients. Tougas et al. (2019) utilized a bioecological approach to synthesize findings in a more updated literature review; however, their framework may not be accessible to everyday educators unfamiliar with highly academic concepts.

The purpose of the present systematic literature review was to build on the findings by Savina et al. (2014) and Tougas et al. (2019) through applying systematic research methods to provide an accessible real-world framework and template for practitioner use. Better outcomes can be achieved for students with mental health needs after discharge from acute psychiatric hospitalization provided the necessary resources are accessible to those outside of academia (e.g., school personnel, caregivers). Having clinically relevant tools also allows for increased collaboration across stakeholders. Finally, summarizing the most recent literature ensures that work in this area is based on current findings that includes wider perspectives, the targeted setting, and methods that are replicable.

## Methods

This systematic review followed the Preferred Reporting Items for Systematic Reviews and Meta-Analyses (PRISMA) reporting guideline recommendations (Moher et al., 2009). The PRISMA 27-item checklist and four-phase flow diagram (see below) were used to ensure the integrity and rigor of data extraction and reporting.

### Protocol

Methods of review and inclusion criteria were specified in a research proposal that was reviewed for feasibility, a priori, by all authors. Keywords and search terms were generated by the second author (JF); however, all authors provided input to generate the final list prior to database searching. All authors are qualified: Four authors (SM, BW, AT, SM) are currently or have previously been educators on pediatric acute psychiatric inpatient units; one author (JF) is a licensed clinical psychologist with expertise in pediatric psychiatric interventions.

### Eligibility Criteria

Articles retained for the final analysis met the following criteria: (a) peer-reviewed research published in academic journals (i.e., qualitative or quantitative designs, literature reviews) or grey literature (e.g., theses/dissertations, theoretical papers, white papers, technical papers, newsletters, government documents); (b) focus on school-aged youth (ages 6–18 years old); (c) focus on youth diagnosed with a mental health condition who are/were admitted to an acute psychiatric inpatient unit; (d) inclusion of some aspect of transitioning youth post-discharge to school/academic routines; and (e) published in English. As a result, articles were excluded if they focused on (a) youth with medical needs; (b) a medical admission for youth with a mental health diagnosis; and (c) post-discharge transition to a non-school setting. No other data restrictions or publication status specifications were applied.

### Information Sources

#### Search Strategy

An initial literature search was conducted in late October 2019 using PsychINFO, Ovid MEDLINE, PubMed, SCOPUS, Academic Search Premier, EMBASE, and ERIC bibliographic databases. Keywords used involved a combination of Medical Subject Headings (MESH) and free-text terms. All MESH search terms were expanded to ensure a broad search. The following search term phrasing was used across databases: [(inpatients or hospitalized patients) AND ((mental disorders OR psychiatric unit) OR psychiatry OR psychology)] AND [school AND ((school health services OR transitional care OR school reintegration) OR discharge planning)].

The search was completed and checked by one reviewer (JF) and rechecked by another (SM). Additional articles were included to ensure the completeness of the search. The extra articles were found based upon input from experts and colleagues in the field as well as non-systematic searches using generic databases (e.g., Google, GoogleScholar, EBSCO). These articles were added without verifying whether they were already found using the systematic database search.

A follow-up literature search was conducted in March 2022 to include updated research (i.e., published October 2019 and onward). The same databases and search methods (including keywords) were employed to identify recent articles aligned with our inclusion and exclusion criteria. All other strategies to select and extract data from the recent articles were identical to those described below.

#### Selection of Studies

Once the electronic search was complete, duplicates were removed. Articles were assessed for relevance through a multi-phase screening process. In Phase One, all titles and abstracts were screened, and articles not meeting inclusion criteria were excluded. If it was unclear from the title or abstract if inclusion/exclusion criteria were met, the article was retained. In Phase Two, the full text of all retained articles was reviewed separately by the first and second authors to determine if they met inclusion criteria. Phase Two inter-rater reliability was 100%. In Phase Three, the first (SM) and second (JF) authors hand-searched the reference lists of the remaining articles. The full text of articles identified via hand-searching was read and inclusion/exclusion criteria were used; inter-rater reliability was 100%. In Phase Four, all remaining articles were read for data extraction (see section below). Articles were divided among two author dyads (SM/SM or BW/AT). Articles not meeting eligibility based upon the research defined data-extraction tool were excluded from the final synthesis. Phase Four inter-rater reliability across author dyads was 100% and 100%, respectively.

### Data Extraction

Data were extracted using a spreadsheet developed for this study. Basic article information, including title, year of publication, author, publishing journal, and type (e.g., peer-reviewed article, grey literature), was coded. Next, the assigned author dyad team read each article. Based upon the full-text review, the following data were extracted and synthesized:

1. Barriers or challenges when transitioning youth from acute psychiatric care to school
2. Significance of need or reasons for a transition process
3. Key factors aiding the transition process
  - Student voice (expanded to “Stakeholder Voice” through synthesis)
  - Point person
  - Recommendations or accommodations
  - Transition meeting
  - Other key factors noted upon full-text review
4. Other potentially important information

## Results

### Search Results

**Figure 1:**
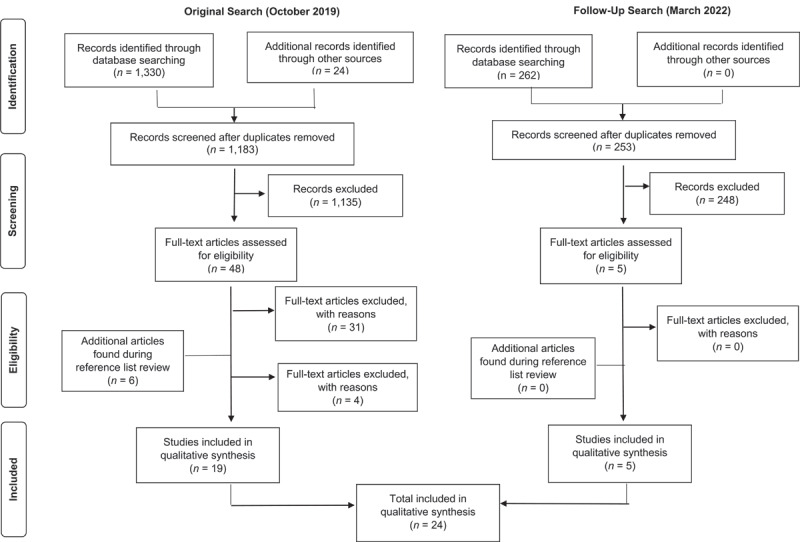
PRISMA Flow Diagram.

The database searches resulted in a total of 1,330 articles (see Figure 1 for PRISMA flow chart). Twenty-four articles were added to ensure inclusiveness of the search. After this was complete and duplicates were removed, a total of 1,183 articles were retained for further analysis. During Phase One, review of the titles and abstracts of the articles yielded a total of 48 articles. During Phase Two, 17 articles were retained after the initial full-text review and 6 additional were identified by hand-searching their reference lists. During data extraction, an additional four articles were excluded due to content not being focused specifically on transitions from acute psychiatric settings to school. Thus, a total of 19 articles were retained for qualitative synthesis. Out of these original 19 articles, 11 were empirical peer-reviewed studies, 3 were literature reviews, and 5 were considered grey literature. After the updated search in March of 2022, five more articles were found that met criteria and were added to the list. Three were empirical peer-reviewed studies; two were grey literature (including one dissertation). Most of the articles were recently published; 19 of the total 24 were published in 2010 or later, two were published between 2000 and 2010, and three were published between 1964 and 1992.

### Student Perspective

Ten articles highlighted youth perspectives during or after their transition back to school. Outcomes suggest that students need increased emotional and academic support. Youth experienced elevated levels of anxiety after discharge in the areas of peer relationships (88%), followed by coping skills, academic performance, and school staff relationships (Weiss et al., 2015). Preyde et al. (2017) found that 32.3% of youth changed schools because of reintegration difficulties. Marraccini et al. (2022) categorized transitioning students’ self-reported areas in need of support into social-emotional experiences, academic experiences, and parent engagement. Thus, the lack of support during the transition period affects students’ immediate academic performance and has widespread implications.

### Caregiver Perspective

Five articles highlighted caregiver perspectives. Caregiver-specific themes included desire for stronger hospital-to-school transition processes; elevated levels of caregiver fatigue, stress, and strain; and anxiety for their child’s future. Caregivers with sole responsibility for their child’s transition back to school experienced high levels of distress (Blizzard et al., 2016; Tisdale, 2014). Articles suggest that the absence of formalized transition processes places increased responsibility and burden on caregivers. Given that the family unit is in crisis, it is unreasonable to assume that caregivers can coordinate educational plans for their child, especially as caregivers likely have no formal training in education or mental health.

### School Personnel Perspective

Six articles focused on school personnel perspectives. Across articles, common relevant themes included an overall acknowledgment of the need for a supportive process, a desire to help, and a lack of formal processes or training to implement a dedicated transition system. In a survey of school staff, only 16% claimed to have a formal transition process in place (Marraccini et al., 2019). However, when schools implemented informal methods to aid reintegration post- discharge, including check-ins with staff (Center for Mental Health in Schools at UCLA [CMHS], 2014; Tisdale, 2014; Tougas et al., 2019) and/or a slower school transition (Clemens et al., 2011; Marraccini et al., 2019; Preyde et al., 2017; White et al., 2006; White et al., 2017), students had improved outcomes. Thus, even the adoption of simple measures and methods can be helpful.

### Hospital Personnel Perspective

Four articles included hospital personnel perspectives (see Table 1). Of these articles, three focused on rehospitalization concerns and/or lack of structured transition processes. Further, hospital personnel noted that the absence of or errors in communication between the psychiatric hospital/acute setting and the school of record contribute to increased rehospitalization risk (CMHS, 2014). Given the high level of work acute psychiatric unit staff employ to support their patients, improved communication between psychiatric hospital and school settings needs immediate attention.

**Table 1:** Data Extraction Categories Across Retained Articles (N = 24).


	TYPE OF ARTICLE	PERSPECTIVES	TRANSITION BARRIERS	SIGNIFICANCE OF NEED	POINT PEOPLE	STAKEHOLDER VOICE	RECOMMEN-DATIONS	TRANSITION MEETINGS	OTHER

Blizzard et al., 2016	Research Article	X^b,c^	X^a,d^	X^d^	X	X^a,b,d^	X	X	

Clemens et al., 2010	Research Article		X^a^		X	X^a,c^	X		X

Clemens et al., 2011	Research Article		X^b,d^	X^b,d^	X	X^a,b,^		X	X

Hall & DuBois, 2020	Grey Literature			X^c^	X	X^a,b,c^	X	X	X

Iverson, 2017	Grey Literature (Dissertation)	X^a^	X^a, c^	X^c^		X^a,c^	X	X	

Johnson & Rubin, 1964	Research Article	X^c^					X		

Loeper, 2021	Grey Literature (Dissertation)	X	X^a, b, c, d^	X	X^d^	X^a,b,c,d^	X	X	X

Marks, 1987	Research Article	X^b,c^	X^a, b, d^	X^b, d^	X	X^a,b,d^	X	X	X

Marraccini et al., 2019	Literature Review	X^c^	X	X	X	X^a^	X	X	X

Marraccini & Pittleman, 2021	Research Article	X^a^	X^a, c^	X	X^c^	X^a, b, c^	X	X	X

Marraccini et al., 2022	Research Article	X^a,b,c,d^	X	X		X^a,b,c,d^	X	X	X

McLeer et al., 1992	Research Article	X^a,c^				X^c,d^	X		

Preyde et al., 2017	Research Article					X^a^	X		X

Preyde et al., 2018	Research Article	X^a^	X^a,^		X	X^a^	X		

Preyde et al., 2021	Research Article	X^a^	X	X		X^a^	X		X

Savina et al., 2014	Literature Review	X^a,d^	X^b, d^	X^b, d^	X	X^a,c,d^	X	X	X

Simon & Savina, 2005	Research Article						X	X	

Simon & Savina, 2010	Research Article		X^c,d^	X^c,d^	X	X^a,b^	X	X	X

Center for Mental Health in Schools at UCLA, 2014	Grey Literature	X^d^	X^c,d^	X^c,d^	X	X^b^	X	X	

Tisdale, 2014	Grey Literature (Dissertation)	X^b^	X^b,d^	X^b,d^	X	X^a^	X	X	X

Tougas et al., 2019	Literature Review	X^a^	X	X		X^a^	X	X	X

Weiss et al., 2015	Grey Literature	X^a,b^	X^b^	X^b^	X	X^a,b^	X	X	X

White et al., 2006	Research Article	X^a,d^	X^d^	X^d^	X	X^b,c^		X	X

White et al., 2017	Grey Literature		X^d^	X^d^	X	X^a^	X	X	X


*Note*: a = student; b = caregiver; c = school; and d = hospital.

### Barriers/Challenges When Transitioning Patients From Acute Psychiatric Care to School

Barriers and challenges exist for successful school transitions after an acute inpatient psychiatric admission. Existing barriers are best interpreted through the lens of specific stakeholders (e.g., student, caregiver, school staff, hospital). Further, a general and overarching barrier in fostering a successful school transition is limited interdisciplinary collaboration and resource sharing between education and medical systems (CMHS, 2014; Savina et al., 2014; Simon & Savina, 2010).

#### Student Barriers

Seven articles highlighted student barriers in the transition process. Specific concerns from transitioning students were overall anxiety about returning to school and difficulty experiencing/managing mental health symptoms in school (Blizzard et al., 2016; Clemens et al., 2010, 2011; Iverson, 2017; Marks, 1987; Marraccini & Pittleman, 2022; Preyde et al., 2018). Overall student investment in recovery impacts successful transitions (Blizzard et al., 2016; Clemens et al., 2010, 2011; Iverson, 2017; Marks, 1987; Preyde et al., 2018). Specifically, if the youth is uninterested or unmotivated to commit to mental health treatment upon discharge, their transition back to school will be less successful.

#### Caregiver Barriers

Five articles addressed caregiver transition barriers. Across these articles an interesting outcome was noted: Caregiver attitude, knowledge, and behavior diminish the success of their child’s return to school. Further, the school transition was negatively impacted when caregivers had less investment in their child’s recovery or had low expectations of treatment (Clemens et al., 2011; Tisdale, 2014). Low caregiver investment or expectations may stem from mixed feelings, including guilt, or from concerns about post-hospitalization stigma (Savina et al., 2014; Weiss et al., 2015). As a result, caregivers may be reluctant to sign consent or release of information for the school and hospital to connect, preferring to handle the sharing of information themselves (Marks, 1987). Finally, caregiver knowledge of available resources may create a gap in success between those well versed and those less aware (Clemens et al., 2011; Tisdale, 2014).

#### School Staff Barriers

Across four articles highlighting school staff transition barriers, a lack of focused training was a common concern (CMHS, 2014; Iverson, 2017; Simon & Savina, 2005). In schools where explicit training or ability related to supporting transitioning students with mental health needs were not a priority, results were diminished. Further, the educational climate of the school, as well as educator perceptions of student behaviors upon return to the classroom can impact transition success (Marks, 1987; Marraccini & Pittleman, 2022).

#### Hospital Barriers

Nine articles focused on hospitals barriers in school transitions. First, most types of health insurance do not explicitly cover school transition planning. By not being able to claim reimbursement for school transition planning, clinical teams are limited from committing time and resources for even an informal transition process (Blizzard et al., 2016; Tisdale, 2014). As shortened lengths of acute psychiatric hospitalization stay have become the norm (from 11–44 days to 5–7 days; Clemens et al., 2011), there is little time for effective and collaborative coordination (Blizzard et al., 2016; White et al., 2017), especially when legal/confidentiality considerations such as HIPAA exist (Marks, 1987). Students spend less time out of school once discharged from the hospital (Clemens et al., 2011; White et al., 2017), which further limits the amount of time for communication between stakeholders (CMHS, 2014; Savina et al., 2014; Simon & Savina, 2010) and reduces the ability to develop, implement, and transition individualized supports (White et al., 2017; Clemens et al., 2011). Finally, the need for more outpatient mental health supports was noted (Tisdale, 2014; White et al., 2006). When a student meets discharge criteria, arranging ongoing outpatient mental health services is often difficult. For example, the challenges of securing an outpatient mental health appointment within a reasonable post-discharge time frame can result in a disjointed or ineffective level of care that impacts the school transition.

### Transition Coordinator/Point People

There is strong support for a specific transition point person or coordinator (Blizzard et al., 2016; CMHS, 2014; Hall & DuBois, 2020; White et al., 2006). Across 16 articles, a point person was noted as critical for communicating student needs and/or translating information between the medical and educational worlds. This individual takes the lead on navigating the transition planning and implementation. Ideally, the coordinator begins transition planning prior to discharge from the hospital (Clemens et al., 2010; Clemens et al., 2011; Marks, 1987; Simon & Savina, 2005). For example, Blizzard et al. (2016) indicate that a school transition specialist, a role specifically designated within a pediatric acute psychiatric unit, serves as the main transition coordinator. In this pre-determined transition coordinator role, the individual bridges communication between the hospital and school and facilitates collaboration among stakeholders to develop a plan that addresses the strengths and needs of families and youth.

There is no consensus in the literature on whether the transition coordinator, or lead point person, is best selected from the hospital or the school setting. Loeper (2021) suggested that hospital-based occupational therapists are ideal to serve as transition coordinator, given their expertise in teaching coping skills and developing environmental accommodations. Others list various hospital and school-based employees, such as school psychologists or social workers, as options to consider for a point person. Regardless of who fills the point person role, their main and most important responsibility is facilitating collaboration between the home school and the acute psychiatric hospital to ensure a successful transition. As ensuring clear communication is critical, we suggest that both a hospital point person and a school point person be identified, with one of them being the central transition coordinator.

#### Hospital/Medical Point Person

The ideal or standard is having a dedicated hospital role for a primary point person leading hospital-to-school transitions (Savina et al., 2014). This individual is a mental health professional who coaches teachers on setting up realistic short- and long-term expectations for the student and provides accommodation recommendations. If having a dedicated school transition lead position is not feasible or if dedicated resources are unavailable, others who could fulfill the hospital point person role would be medical providers, social workers or psychologists, occupational therapists, or an as-needed school transition specialist or school-based mental health therapist (Blizzard et al., 2016; Loeper, 2021; Savina et al., 2014; Weiss et al., 2015). Regardless of who the point person is, they should be able to speak to treatment needs and supports that can translate to a school setting.

#### School Point Person

Although the school point person is critical, there is no consensus on who would be the best fit for this role. Rather, outcomes suggest that focusing on what is needed for the transitioning youth and then identifying the best fit within the school may be the best option. Individuals most often fulfilling the school point person role include school psychologists (Marraccini et al., 2019; Savina et al., 2014; Tisdale, 2014), followed by social workers (Savina et al., 2014; Tisdale, 2014; White et al., 2006), school counselors (Savina et al., 2014; Tisdale, 2014), district-wide clinical coordinators (Savina et al., 2014), the youth’s special education teacher (Simon & Savina, 2010), school nurses (Tisdale, 2014), and adjustment counselors (Tisdale, 2014). Regardless of who fills the school point person position, a common theme across articles was the importance of identifying someone who could actively and effectively communicate student and school staff needs and collaborate with the hospital point person/staff.

Main roles and responsibilities of the school point person may include translating treatment goals to educational accommodations (Clemens et al., 2011; CMHS, 2014; White et al., 2017), providing support to the student and caregiver(s), facilitating the student’s ability to voice their concerns, and supporting the student’s involvement in coordinating school supports (Clemens et al., 2011). The school point person should also counsel the student on how to address peers when asked where they have been (Marks, 1987; Preyde et al., 2018; White et al., 2017) and provide the student a safe outlet when help is needed to support coping skills use (Clemens et al., 2011; White et al., 2017). Finally, the school point person must coordinate with teachers and school staff to create a feasible plan for completing make-up work and support overall educational success (CMHS, 2014; White et al., 2017).

### Incorporating Key Stakeholder Voice

Incorporating student voice was one of the predetermined categories necessary in building a successful transition framework from acute psychiatric settings to schools. However, across 22 articles, outcomes revealed the need to expand this concept into “Key Stakeholder Voice.” This new category indicates that there are four key stakeholders whose input is essential to create effective and individualized back to school transition plans: student, caregiver, hospital, and school.

#### Student Voice

Student voice was noted across 18 articles (see Table 1) as important in creating individualized transition plans. Overall, the theme of student voice may be divided into three main categories: academic, social/relational, and emotional.

##### Academic

Students are concerned about their academics upon returning to school (Blizzard et al., 2016; Clemens et al., 2010; Marraccini & Pittelman, 2021; Preyde et al., 2018; Preyde et al., 2021; Weiss et al., 2015; White et al., 2017). Students report distress related to making up missed work while hospitalized and about their overall academic standing.

##### Social/Relational

Students are concerned prior, during, and after the transition process about their social life and peer relationships (Blizzard et al., 2016; Clemens et al., 2011; Iverson, 2017; Marraccini & Pittleman, 2022; Preyde et al., 2018; Preyde et al., 2021; Simon & Savina, 2005; White et al., 2017). Student social/relational concerns include worries about mental health stigma and hospitalization negatively effecting friendships (Blizzard et al., 2016; Clemens et al., 2010; Marraccini & Pittleman, 2022; Weiss et al., 2015; White et al., 2017); explaining absences to peers (Clemens et al., 2010; Marraccini & Pittleman, 2022; Preyde et al., 2018 Preyde et al., 2021); navigating pre-existing social life (Clemens et al., 2010); and relationships with school personnel (Blizzard et al., 2016; Weiss et al., 2015).

##### Emotional

The third student-voiced concern category consists of worry about navigating emotions at school post-hospitalization (Clemens et al., 2010, 2011; Marraccini & Pittleman, 2022; Preyde et al., 2018; Preyde et al., 2021; Weiss et al., 2015). Students with mental health needs already have difficulty regulating their emotions at school prior to hospitalization. Therefore, it is logical that they would be concerned about their personal coping skills amidst their return to school.

#### Caregiver Voice

The importance of caregiver voice was noted across 11 articles (see Table 1) in informing transition planning (Blizzard et al., 2016; Clemens et al., 2011; CMHS, 2014; Simon & Savina, 2005; Weiss et al., 2015; White et al., 2006; White et al., 2017). Including caregivers in developing their child’s transition plan supports those who are emotionally distressed due to their child’s hospitalization and mental health difficulties. Further, caregivers can provide invaluable information regarding needed supports for their child in areas of social-emotional functioning, learning, access to increased school services, and advocacy (Marks, 1987; Simon & Savina, 2005; Weiss et al., 2015). Other reasons for including caregiver voice include the importance of proactively providing a clear description of the transition process, assessing their level of understanding, allowing them to indicate what they want shared with their child’s school, and completing a release of information (Simon & Savina, 2005).

#### Hospital Voice

Across six articles, the hospital or treatment team emerged as a key stakeholders’ voice. Acute psychiatric hospital staff are in the best position to use their clinical ability to inform transition accommodations and placement considerations. Utilizing the expertise of a multidisciplinary mental health team assists in providing feedback to schools regarding appropriate educational accommodations and settings based on the student’s mental health diagnosis (Savina et al., 2014). Once hospital staff support the youth in developing a proactive coping plan, they can share the individualized strategies with school (see Student Voice, Emotional Domain).

#### School Voice

Across nine articles, school personnel voices were noted as key in the post-discharge transition. School staff have stated their need for further support; they feel unequipped to fully support the student’s needs while managing difficulties that emerge as the student returns to class (Blizzard et al., 2016; Marks, 1987; Marraccini et al., 2022; Tougas et al., 2019). Transitions are impacted due to available resources and the preparedness of a school (McLeer et al., 1992; Savina et al., 2014), highlighting the need for guidance from and better coordination with the hospital side (Marks, 1987; White et al., 2017).

### Recommended Supports

Twenty-two articles provided specific recommendations and supports (see Table 1). These supports and recommendations were broken down into two sections: formal and informal.

#### Formal Supports

Formal supports are those included in an educational plan (e.g., Section 504 Plan or Individualized Education Plan [IEP]). Many areas exist in the transition process where formal supports are necessary. First, formal educational plans must be created to help students make up work and learning missed during absences while hospitalized (Blizzard et al., 2016; Clemens et al., 2010; CMHS, 2014; Hall & DuBois; Iverson, 2017; Loeper, 2021; Marraccini et al., 2019; Preyde et al., 2018; Tougas et al., 2019; White et al., 2006; White et al., 2017). Formal updates on the student’s learning may be needed as a result of their mental health diagnosis, including creating or updating of IEPs, Section 504 Plans, or other placements (Blizzard et al., 2016; Clemens et al., 2010; Iverson, 2017; Johnson & Rubin, 1964; Marks, 1987; McLeer et al., 1992; Preyde et al., 2017; Preyde et al., 2018; Savina et al., 2014; Simon & Savina, 2010; Tougas et al., 2019; White et al., 2017).

Formal recommendations may be necessary to address the emotional aspect of returning to school. Returning students need individualized coping skill plans and treatment recommendations to assist with reintegration stress. Individualized coping plans can be added to a formalized education plan (Blizzard et al., 2016; Clemens et al., 2010, 2011; Marks, 1987; Marraccini et al., 2019; McLeer et al., 1992; Preyde et al., 2017; Preyde et al., 2018; Savina et al., 2014; Simon & Savina, 2005; Weiss et al., 2015; White et al., 2017). Finally, a discharge summary from the medical team can inform formal accommodations (Hall & DuBois, 2020; Simon & Savina, 2005, 2010).

#### Informal Supports

Informal (i.e., unofficial, undocumented) supports are also needed for students transitioning to school post-discharge from an acute psychiatric hospitalization. An initial area where informal supports should be considered is interpersonal relationships with peers and school personnel (Blizzard et al., 2016; Iverson, 2017; Marraccini et al., 2019; Marraccini et al., 2019; Preyde et al., 2017; Preyde et al., 2018; Savina et al., 2014; Simon & Savina, 2005; Weiss et al., 2015; White et al., 2017). When implementing social supports, a strengths-based approach should be utilized (Clemens et al., 2010; CMHS, 2014; Marks, 1987). Informal check-ins with a point person during the first few weeks should be used to monitor student progress and identify additional areas where formal or informal supports are needed (CMHS, 2014; Hall & DuBois, 2020; Tisdale, 2014; Tougas et al., 2019). Other areas where informal supports may be necessary include increasing staff preparation and confidence for the student upon return (Savina et al., 2014; Simon & Savina, 2005), keeping the school point person informed of mental health follow-up (Tougas et al., 2019; White et al., 2017), and ensuring caregivers know who they can contact if they have questions (Weiss et al., 2015).

### Transition Meetings

Before a youth returns to school, there is a need for a transition meeting with key stakeholders to allow coordination of all parties (see Table 1; *n* = 18 articles). Where the meeting occurs and who is present varies (Clemens et al., 2011; White et al., 2017). Those present at the meeting (whether in person, phone, or virtually) should include, at minimum, the student, caregiver, school point person, hospital point person, and outpatient therapist (Clemens et al., 2011). Other school personnel who may also be necessary include nurse, teachers, counselors, and/or school-based therapist.

### Other Key Factors/Considerations

Additional key factors that should be considered when planning for the transition back to school were noted across 17 articles.

#### Overall Staff Training

School staff feel underprepared and undertrained to meet the needs of students with mental health conditions. Despite wanting to support transitioning students, schools are often limited in resources when it comes to training (Marks et al, 1987; Savina et al., 2014; Tisdale, 2014; Weiss et al., 2015; White et al., 2017). When school staff receive structured training in transitioning students after acute psychiatric hospitalizations and training in creating a supportive school culture, schools improve in their ability to provide systematic supports and coordinated resources (White et al., 2006).

#### Timing

The timing of the transition should be thoughtfully considered. A slower transition into school may be ideal for some students (Clemens et al., 2011; Marraccini et al., 2019; Preyde et al., 2017; Weiss et al., 2015; White et al., 2006; White et al., 2017). The most crucial time to reestablish the student in school is the first few days to the first few weeks post-hospitalization (Simon & Savina, 2010). Therefore, timing of the transition based upon student needs may aid in establishing successful outcomes.

#### Family Support

Providing programs and resources to students and their families creates positive transition outcomes (Marks, 1987; Tisdale, 2014; Tougas et al., 2019; Weiss et al., 2015; White et al., 2006). Families feel ill prepared to be responsible in assisting school transitions. However, when family supports or peer-to-peer (student/family) programs are in place, students and their families feel more supported (Blizzard et al., 2016; Weiss et al., 2015).

#### Dedicated Programs

Sites with dedicated programs focusing on acute psychiatric hospitalization to school transitions have better outcomes (Marraccini et al., 2019; Weiss et al., 2015; White et al., 2006; White et al., 2017). With regard to the two models described previously, in the School Transition Program, three positive outcomes occurred: greater caregiver satisfaction, decreased length of inpatient stays, and decreased readmission rates (Weiss et al., 2015). Further, central to the BRYT program’s successful outcomes of increased rates of school attendance, graduation, and day-to-day functioning are core features of providing dedicated spaces for returning students, flexible transition plans, and significant wrap-around support (White et al., 2017).

## Discussion

Goals of this literature review were to provide an updated theoretical framework and clinical template to facilitate successful school transitions for youth after acute psychiatric hospitalization. Our results align with and extend those of Savina et al. (2014) and Tougas (2019) by adding critical key elements, a practitioner lens, and a tangible resource. Four critical elements were identified: involving key stakeholders (student/patient, caregiver, psychiatric staff, and school staff) to understand their perspectives; identifying a single point person within the psychiatric staff and school staff; planning and facilitating a transition meeting; and creating formal and informal supports.

Although systematic protocols and PRISMA guidelines were used, some limitations still exist. First, articles were reviewed through the lens of a hospital educator. Thus, the reviewers have direct experience with transitions from acute psychiatric settings to school; yet, “real-world” interpretation bias may still exist despite taking precautions to guard against doing so. Second, literature in this area is scant. We relied on articles that often had small sample sizes or had less rigorous methodologies, which might have limited the robustness of outcomes. Future work should utilize more rigorous research designs.

Regardless of this review’s limitations, valuable information can be gleaned. First, outcomes suggest that there is not one specific way to successfully transition youth from an acute psychiatric unit back to their home school. Rather, multiple ways exist involving a combination of many different people, individual characteristics or case-specific nuances, and varied setting-specific supports. This highlights the importance of focusing on the overarching characteristics and key elements of supportive transitions, while allowing flexibility to honor the realities within each specific setting.

### Significance of Need, Barriers in Transition Planning

Numerous challenges exist, delaying an ideal acute psychiatric hospital to school transition. Navigating the path of a multisystem continuum of support is complex. However, all stakeholders are searching for the same goal that cannot wait: a more supported transition. The available interdisciplinary research points to many problematic outcomes due to limited formal hospital-home-school transition planning currently being practiced (McLeer et al., 1992; Marks, 1987; Preyde et al., 2017; Preyde et al., 2018; Savina et al., 2014; Tougas et al., 2019; Weiss et al., 2015; White et al., 2006). Rehospitalization risk for youth remains high (Clemens et al., 2010; CMHS, 2014; Preyde et al., 2018; Savina et al., 2014; Simon & Savina, 2005; Tougas et al., 2019; Weiss et al., 2015; White et al., 2017; White et al., 2006), and the prognosis for those who have been hospitalized for inpatient psychiatric care is riddled with ongoing mental health needs and school-related concerns (Johnson & Rubin, 1964; Marks, 1987; Marraccini et al., 2019; Preyde et al., 2018; Tisdale, 2014). Students need specific plans in place to feel supported and capable of success. Caregivers need clear explanations and willing, caring partners to help them navigate complex processes like insurance claims, 504 or IEP evaluations, and school-based mental health care. Schools need ongoing professional development focused on the prevalence of mental illness in school-aged youth, effective tools and interventions, and the development of hospital-home-school transition planning administrative processes. Finally, psychiatric staff need a straightforward solution to support transitions without having to dedicate already scarce resources to non-reimbursable services.

### Stakeholder Voice

Outcomes show that a supportive and successful transition includes the perspectives of multiple stakeholders. Students, caregivers, school personnel, and hospital personnel are all needed to provide insight on the student’s areas of concern, strengths, and educational plan. It is imperative stakeholders work collaboratively to inform next steps in the transition. Without one of these key stakeholders involved, predicted success of the transition plan will lessen. However, involving whoever is available is a crucial step towards the ideal transition until sound relationships and norms can be established.

Caregivers desire support in navigating the transition process (Clemens et al., 2011; Weiss et al., 2015). It is important that parents understand the importance of the transition and of sharing of information between the hospital and school teams early in their child’s hospitalization. The legal guardian must sign a release of information (Simon & Savina, 2005); schools and/or hospitals should have these forms prepared and ready to discuss with caregivers. Understanding caregivers’ concerns about their child’s school can assist in identifying areas to target when transition planning. The hospital and school point persons should quickly establish who will lead the process of keeping caregivers informed of updates.

Transition plans should incorporate plans to support the student’s academic, social/relational, and emotional concerns. For student academic concerns, for example, having a concrete plan for make-up work during the transition process helps in reducing transition distress (Clemens et al., 2010; Weiss et al., 2015). If social/relational concerns exist, the hospital point person can role-play scenarios with the youth during admission; the school point person, in turn, can set up creative ways to support these interactions post-discharge (Preyde et al., 2017). For emotional concerns, youth should work towards understanding their triggers and identify coping skills. Mental health professionals can assist by establishing a coping skill plan with the youth at discharge and re-entry to help them identify emotions, understand the impact of these emotions, and practice various ways of coping (Clemens et al., 2010; Preyde et al., 2018).

Hospital stakeholder voice may be the most difficult perspective to gain. Many factors may contribute to the absence of the hospital stakeholder, including nonbillable service rendering, limited availability of key medical providers and/or mental health providers, and possible limited knowledge of providers on specific educational implications of psychiatric diagnoses. While these barriers may limit the collaborative nature of the stakeholder team and negatively impact the success of the transition plan, there are alternatives. For example, school personnel can consider including the voice of the student’s outpatient mental health therapist or a school-based mental health professional to give clinical insight. It may also be helpful to request the patient’s discharge summary from the caregiver.

For school personnel voice, the greatest area of need involves supports or training to facilitate gaining knowledge on acute psychiatric admission to school transitions (Blizzard et al., 2016; Marks, 1987; McLeer et al., 1992; Savina et al., 2014). School personnel have all but yelled from the rooftops that they need guidance, particularly from mental health professionals and hospital staff. They want effective coordination between the school and the mental health team (Blizzard et al., 2016; Marks, 1987; Simon & Savina, 2010; White et al., 2017). Without proper preparation for school re-entry, students may exhibit disruptive behaviors (Blizzard et al., 2016; Tougas et al., 2019). Further, the transition team should solicit all stakeholder input and carefully consider expectations and resources to identify possible gaps. Unrealistic expectations are often set for schools that are not possible given current resources. Brainstorming creative solutions with the transition team can help in ensuring the student’s needs are met and supports are realistic.

### Transition Coordinator and Point People

A key point consistently supported was the importance of identifying a specific transition coordinator to mediate between the student, caregivers, school, and hospital staff. Caregivers report that transitioning their child to school on their own can be difficult and frustrating; not surprisingly, therefore, relying on caregivers to navigate the process alone yields unsuccessful outcomes (Blizzard et al., 2016; Hall & DuBois, 2020; Johnson & Rubin, 1964; Marks, 1987; McLeer et al., 1992; Tisdale, 2014; Weiss et al., 2015). Identifying a specific point person can alleviate caregiver stress and burden. The point person has many responsibilities, but their main purpose is to connect the school, caregivers, student, and hospital to create a plan for moving forward with the school transition.

An ideal transition would have a specific point person on both the school and the hospital side (Blizzard et al., 2016; Clemens et al., 2010, 2011; CMHS, 2014; Hall & DuBois, 2020; Iverson, 2017; Marks, 1987; Marraccini et al., 2019; Savina et al., 2014; Simon & Savina, 2010; Tisdale, 2014; Weiss et al. 2015; White et al., 2006; White et al., 2017), with one being elected as the ultimate transition coordinator. The purpose of these roles is to communicate accurate information between the two settings. Our outcomes listed many roles, responsibilities, and persons that could serve as the point person. In the end, the main thing is that point person is available and able to coordinate and communicate stakeholder voices.

Relying on the student voice to identify the school point person or support persons may be necessary to ensure a positive transition outcome. The expectation that anyone can fill this role is false. The student needs to feel safe and supported; if rapport and relationship are not established with the school point person, the plan will not be implemented successfully and the student will continue to feel unsupported (CMHS, 2014; Marks, 1987; McLeer et al., 1992; Marraccini et al., 2019; Savina et al., 2014). The school support person should have a clear understanding of expectations and attitudes that need to be present to support the student. The last item to consider is time. The person in this position needs to be able to have adequate time to support the student (White et al., 2017).

### Recommendations/Accommodations

It is critical that both formal and informal supports are in place for transitioning students. The principal areas where students need supports are academic, social, and emotional. The types of accommodations that will create a safe and appropriate school environment can be developed via student, caregiver, hospital staff, and school staff feedback. This may include creating a safety plan and/or coping skills plan for school, determining if the school is an appropriate fit, and making changes to the student’s access to educational learning. The hospital point person can provide the school point person with relevant medical information, including coping skills plans to translate into a Section 504 Plan or IEP accommodations, and informal supports that align with mental health treatment. A process like this supports each stakeholder and ensures goals targeting the student’s mental health and education are aligned.

### Transition Meeting

A comprehensive transition meeting should occur before the student returns to school. Persons involved in the meeting should include as many of the stakeholders as possible, including caregiver(s), school personnel, counselor, school nurse, school psychologist, hospital representative (e.g., social worker, school transition specialist), and (if age-appropriate) the student. Starting communication early in the hospitalization to understand family/student needs and existing school issues ensures that there is adequate time to develop a transition plan that encompasses all of the needs, abilities, and resources of all stakeholders, and that any ancillary needs (e.g., changing educational plans, training staff, obtaining additional services/resources) are addressed before school reintegration occurs.

### Other Key Factors and Considerations

#### Overall Staff Training

Outcomes suggest that overall, most staff in school settings feel underprepared and uninformed on how to help students experiencing mental health challenges. Such absence of knowledge affects the student’s short-term reintegration success and their long-term educational outcomes. Schools and school districts should consider supporting their teachers by providing targeted training and resources. While school counselors are more adept at managing mental health crises, most schools only employ a few counselors to service all students. Further, school psychologists typically only serve students in special education and, therefore, may be unable to aid students with mental health needs.

Along with lack of mental health training and education for school staff, the timing of the school transition should be considered. Clemens et al. (2010) noted the importance of considering the high likelihood that older students’ ongoing mental health symptoms may be exacerbated by stress resulting from being discharged and transitioning back to school. Thus, it is imperative to support successful educational and mental health outcomes (Blizzard et al., 2016; Iverson, 2017; Marraccini et al., 2019; Marraccini et al., 2022; Preyde et al., 2017; Preyde et al., 2018; Savina et al., 2014; Simon & Savina, 2005; Weiss et al., 2015; White et al., 2017). A well-timed transition plan and formal and/or informal supports can increase the likelihood that the student is able to make up missed time and assignments, and can continue making progress in their academics and mental health treatment.

All stakeholder voices should inform the timing of the transition while balancing the student’s personal, treatment, and educational needs. A slow and systematic reintegration back to school can lessen the impact of the transition for certain students (e.g., those with significant school stressors). Options for slower transitions include the student starting on a half-day schedule for the first few days, spending the first few days in the guidance office working on make-up work, or increasing class-by-class participation as they feel prepared.

#### Caregiver Support

Caregivers of students who need acute mental health care are typically underprepared and overwhelmed when their child returns home. Caregivers note that they feel more successful when they can access resources and support programs in their community; however, such programs can be difficult to access. Caregivers’ needs have a direct impact on their child’s educational and transitional success long-term. As a result, addressing caregiver needs should be part of the transition framework.

#### Dedicated Programs

Hospitals or schools that have dedicated resources to support transitions back to school post-discharge from acute psychiatric units have improved short- and long-term outcomes (Weiss et al., 2015; White et al., 2006; White et al., 2017). The BRYT (White et al., 2006; White et al., 2017) and School Transition Program (Weiss et al., 2015) are two dedicated acute psychiatric hospitalization to school transition programs that may be replicated. However, as many schools and hospitals are unable to make this type of commitment, research supports that even informal implementations for transitions encourages better outcomes (Marraccini et al., 2019).

## Conclusion

Our hope is that these findings and the proposed framework may serve as a basis for further research. More work must occur to identify gaps and to make improvements. In the meantime, this article supplies a tangible resource and roadmap for practitioners when transitioning patients from acute psychiatric care units to school.

Developing standard practices for transitioning youth from acute psychiatric hospitalization to their home school will require practitioners, fluent in hospital and school cultures, collaboratively implementing shared practices. Doing so is critical to establishing a foundation of observable and measurable data to foster the pursuit of evidence-based best practices.

It is understandable that educators and psychiatric units may find providing this level of support difficult depending on their program’s current resources. However, our outcomes are meant to provide the critical elements for an ideal transition process from an acute psychiatric unit back to school. Although obtaining an ideal transition may not be presently possible, making progress, even incrementally, towards an ideal transition shows promise. Transitions between acute psychiatric units and schools are difficult and need standardization. It is time to begin using common language and goals, collaborating between hospital and school, and developing best practices within the field – our students need us.

## Additional File

The additional file for this article can be found as follows:

10.5334/cie.61.s1 Supplementary File 1. School Transition Plan Template.
