# Supplementary material for: Supportive Transition Planning for Adolescents Transitioning From Psychiatric Hospitalization to School: A Systematic Literature Review and Framework of Practices
Source: Contin Educ. 2023 Jan 30;4(1):23–40. doi: 10.5334/cie.61 (PMC11104309; doi:10.5334/cie.61)
Supplement: Supplementary File 1. — School Transition Plan Template. [file cie-4-1-61-s1.pdf]

School Transition Plan Template

Template should be used as a guide and edited to fit needs of practitioners/stakeholders; sample questions within each key factor/category

KEY STAKEHOLDERS/POINT PEOPLE<br>*Determine and fill out as soon as possible after admission to facility*
**STUDENT**<br>• Name:<br><br>• School:<br><br>• Grade:
**PARENT(s)/CAREGIVER(s)**<br>• Name(s):<br><br>• Phone:<br><br>• Email:
**SCHOOL PERSONNEL**<br>• School Transition Point Person (Name and Position):<br><br>• Teachers/School Staff to Be Informed (Names and Positions):<br><br>• Other Supportive Staff to Be Involved (Names and Positions):
**HOSPITAL/MENTAL HEALTH STAFF**<br>• Hospital/Mental Health Point Person (Name and Position):<br><br>• Other Hospital/Mental Health Staff to Be Involved (Names and Positions):
**Transition Meeting**
**TRANSITION MEETING DETAILS:**<br>• Date:<br>• Time:<br>• Location:
**PARTICIPANTS:** *(Should include the student and school transition point person at minimum; each key stakeholder should be invited)*

Midura, S., Fodstad, J., White, B, Turner, A., & Menner, S. (2023). Supportive Transition Planning for Adolescents Transitioning From Psychiatric Hospitalization to School: A Systematic Literature Review and Framework of Practices. *Continuity in Education*

- Invited:
- Attended:

MEETING AGENDA/NOTES (*Sample agenda):*

- Introductions
- Student Voice Review
- Parent/Caregiver Voice Review
- School Point Person/School Staff Review
- Medical Point Person/Hospital Staff Voice Review

STAKEHOLDER VOICE

Recommended for each stakeholder to fill out/relay prior to transition meeting; should inform recommendations/accommodations

STUDENT VOICE (*Recommended to discuss at the hospital with student and hospital staff if possible)*

My Strengths

The class(es) I am strongest in are:

My favorite part(s) about school are:

Two goals I have for the remainder of this school year are:

Academic Planning

The class(es) I am strongest in are:

-
-

Here are my top concerns about my academic work when I return to school:

Social/Relational Planning

When I return to school and someone asks me where I have been, I will say:

(Suggested response if information is not wanted to be shared: "I wasn't feeling well, thank you for asking" and then changing the subject, asking them a question, or stating that you have to speak to your teacher about your missed assignments)

(Recommended: Work with a mental health staff member to create this statement)

Here are my top concerns related to peers and/or staff when I return to school:

A few school staff members I feel the most comfortable with are:

Types of support I could use from my teachers/school staff during my transition back to school would be:

Emotional Planning

On a scale from 1-10, with 1 being the **least** stressed and 10 being the **most** stressed, how would you rate your stress towards returning to school?

1 2 3 4 5 6 7 8 9 10

Coping Skill Plan

(Recommended to work with a mental health professional on this if possible – can inform accommodations/educational plans)

PARENT/CAREGIVER VOICE

Here are my top concerns or questions regarding my child transitioning back to school:

Here are questions I might have about the transition process for my child, if they have not been addressed already:

SCHOOL POINT PERSON/SCHOOL STAFF VOICE

Here are questions/concerns that I have regarding this student's transition to discuss with the medical point person/hospital staff:

Here are recommendations that I would make for this student moving forward at school (informal or formal accommodations):

Midura, S., Fodstad, J., White, B, Turner, A., & Menner, S. (2023). Supportive Transition Planning for Adolescents Transitioning From Psychiatric Hospitalization to School: A Systematic Literature Review and Framework of Practices. *Continuity in Education*

MEDICAL POINT PERSON/HOSPITAL STAFF VOICE

Here are questions/concerns that I have regarding this student's transition to discuss with the school point person/school staff:

Here are recommendations that I would make for this student moving forward at school (informal or formal accommodations):

Recommendations/Accommodations

Recommended to be compiled and discussed prior to transition meeting if possible, but can be finalized at transition meeting

Formal Supports – Medical

Medication management needed at school

- ☐ Yes
- ☐ No

If yes:

Discharging Medication(s)	Dosages	Time(s) and Place of Administration	Prescriber for Follow-Up

Formal Supports – Academic

Academic Make-Up Plan

(*Should be worked out between student and school point person/school staff; school point person should communicate with teachers in order to get this information prior to transition meeting; teachers should consider what work is essential to complete)*

Access to learning: *(Utilize stakeholder voices to inform these concerns of each stakeholder, recommendations, etc.)*

- ☐ Review and revision of IEP or 504 accommodations
- ☐ Evaluation for special education
- ☐ Creation of 504 Plan
- ☐ Breaks from tasks (classroom, work, etc.)
- ☐ Frequent checks for understanding (assignments and directions)
- ☐ Smaller classroom setting
- ☐ Fewer transitions
- ☐ Early or late passing time
- ☐ Expression of knowledge of material individually rather in large class group

Absences: Ensuring absences are covered where possible, planning for future absences, consider supplemental academic support that is needed based on absences

- ☐ Extended time to make up missed work
- ☐ Extended time to make up missed exams or tests
- ☐ Excused absences when student is absent for follow-up care appointments
- ☐ Excused absences for time out of school while hospitalized

Formal Supports – Emotional

Formalized coping skill plan for school: *(Should be worked on between student and therapist, ensure ability to utilize coping skills at school, alert teachers that need to be alerted, considered for 504/IEP accommodations, etc.)*

- ☐ 504 Plan
- ☐ IEP accommodations
- ☐ School safety support plan
- ☐ Communication with teachers regarding plans
- ☐ Frequent check-ins with trusted school staff member
- ☐ Safe space/zone in building where student can go when feeling overwhelmed
- ☐ Access to consistent school support person

Informal Supports – Social Considerations/Supports *(Work with student to develop a plan to support social needs at school)*

Informal Supports – Mental Health Considerations/Supports *(Work with student and mental health staff to create options for the student and their needs)*

- ☐ Access to medication at school via school nurse
- ☐ Safe space/zone
- ☐ Check-in/check-out with trusted adult
- ☐ Other:

Informal Supports – Strength-Based Approach *(Suggestion to focus on and list student strengths, abilities, talents, skills, etc.)*

Transition details: (*Utilize stakeholder voices to determine this; consider that certain literature recommends a slower transition)*

- ☐ Student will return to full schedule
- ☐ Student will return on reduced schedule (partial days)
- ☐ Student will have access to space to complete work

What follow-up looks like: When can the student expect to meet with the School Point Person again? How often will this happen/for how long after the initial transition?

Who should the caregiver(s) contact with follow-up questions and concerns during the transition period?

Midura, S., Fodstad, J., White, B, Turner, A., & Menner, S. (2023). Supportive Transition Planning for Adolescents Transitioning From Psychiatric Hospitalization to School: A Systematic Literature Review and Framework of Practices. *Continuity in Education*
